# Complete Migration of Metallic Plate in a Pseudodiverticulum of Cervical Esophagus

**DOI:** 10.70352/scrj.cr.26-0097

**Published:** 2026-08-19

**Authors:** Jun Onumata, Shinichiro Kobayashi, Tomotaka Takaki, Atsuhiko Iwao, Kazuaki Yokota, Mika Matsukuma, Takahiro Enjoji, Yasuhiro Maruya, Shunsuke Murakami, Makoto Osaki, Susumu Eguchi, Kengo Kanetaka

**Affiliations:** 1 Department of Surgery, Nagasaki University Graduate School of Biomedical Sciences, Nagasaki, Nagasaki, Japan; 2 Department of Surgery, Shuuwa General Hospital, Kasukabe, Saitama, Japan; 3 Department of Orthopedic Surgery, Nitobe Memorial Nakano General Hospital, Tokyo, Japan; 4 Department of Plastic and Reconstructive Surgery, Nagasaki University Graduate School of Biomedical Sciences, Nagasaki, Nagasaki, Japan; 5 Department of Orthopedic Surgery, Nagasaki University Graduate School of Biomedical Sciences, Nagasaki, Nagasaki, Japan; 6 Department of Surgery, Nagasaki Harbor Medical Center, Nagasaki, Nagasaki, Japan; 7 Department of Surgery, Juzenkai Hospital, Nagasaki, Nagasaki, Japan

**Keywords:** esophageal diverticulum, anterior cervical discectomy and fusion, sternocleidomastoid muscle flap

## Abstract

**INTRODUCTION:**

Migration of cervical hardware into the esophagus after anterior cervical discectomy and fusion (ACDF) is a rare but serious complication. Most esophageal injuries present within days of surgery, and delayed diagnosis may lead to perforation and deep-neck or mediastinal abscess. Safe, definitive treatment is only possible with implant removal to prevent mediastinitis, aspiration pneumonia, and sepsis. Herein, we present a case of an uncommon late-onset esophageal complication due to metallic plate migration 20 years after ACDF, which was managed using direct suturing and sternocleidomastoid muscle (SCM) flap reinforcement.

**CASE PRESENTATION:**

A 63-year-old man who had undergone ACDF 20 years previously was admitted to a local hospital with fever, dysphagia, and neck pain. Based on the CT findings, the patient was diagnosed with esophagitis secondary to a foreign body in the cervical esophageal diverticulum. An endoscopic study showed that a metallic plate for the ACDF was exposed in the posterior wall of the cervical esophageal diverticulum. The patient was then transferred to our hospital for surgical treatment. During surgery, the metallic plate was found to be completely exposed to the lumen of the diverticulum of the cervical esophagus. After opening the posterior wall of the esophagus, the metallic plate was successfully removed and the diverticulum was resected. The defect in the cervical esophagus was closed and reinforced using an SCM flap. The patient recovered uneventfully and was discharged after radiologic confirmation of unhindered passage of contrast medium and no leakage. Histological examination of the resected diverticulum wall revealed the mucosa and submucosa, but not the muscular layer.

**CONCLUSIONS:**

Metallic plate migration into a pseudodiverticulum of the cervical esophagus occurring 20 years after ACDF is a rare but serious complication. The metallic plate was removed and the esophageal diverticulum was closed using direct suturing, followed by reinforcement with an SCM flap.

## Abbreviations

ACDF
: anterior cervical discectomy and fusion
SCM
: sternocleidomastoid muscle

## INTRODUCTION

Migration of cervical hardware into the esophagus after ACDF is an uncommon but serious complication in spine surgery.^1–3)^ Clinically, patients with prior ACDF who develop dysphagia, odynophagia, persistent sore throat, neck pain, or halitosis should be evaluated for hardware migration and secondary esophageal injury, including occult perforation.^2)^ Most hardware-related esophageal injuries occur within days after ACDF. When the diagnosis is delayed, morbidity increases with mediastinitis, deep cervical abscess, osteomyelitis, aspiration pneumonia, and sepsis in severe cases.^4,5)^ Ko et al. defined delayed esophageal hardware migration after anterior cervical spine surgery as migration diagnosed more than 30 days after surgery.^6)^ In rare late-onset esophageal complications of ACDF, chronic inflammation due to mechanical factors, such as screw loosening, plate micromotion, and chronic friction against the posterior esophageal wall, can cause erosion, delayed perforation, and deep-neck infections.^7)^ Therefore, safe and definitive treatment is feasible with prompt implant removal and reconstruction of the esophagus. Herein, we describe an unusual and extremely late presentation of esophageal injury due to the migration of a metallic plate 20 years after ACDF, which was successfully treated with implant removal, direct suturing, and SCM flap reinforcement.

## CASE PRESENTATION

**Fig. 1:**
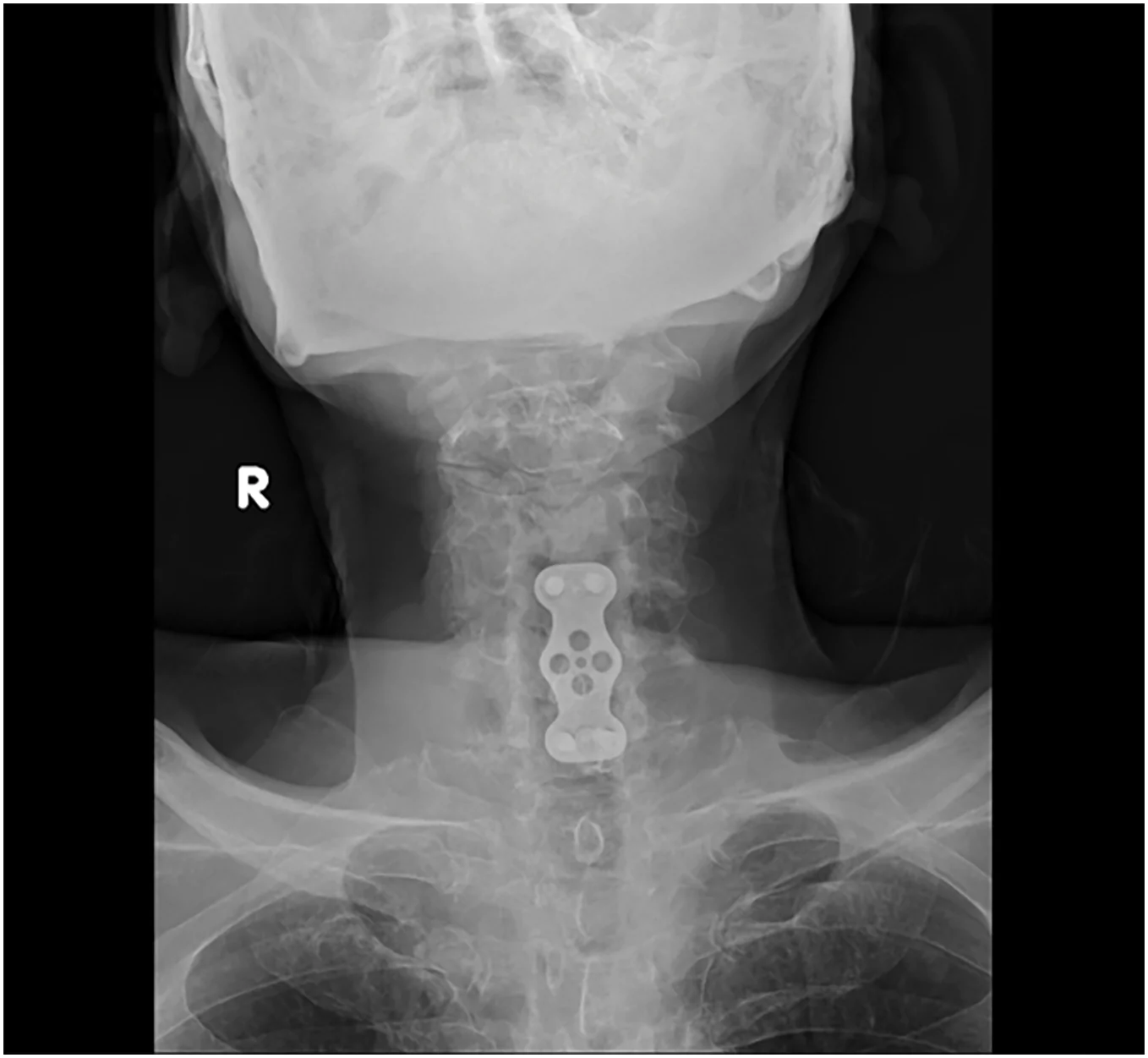
Radiographic examination of the neck revealed a metallic plate fixed to the cervical spine.

**Fig. 2:**
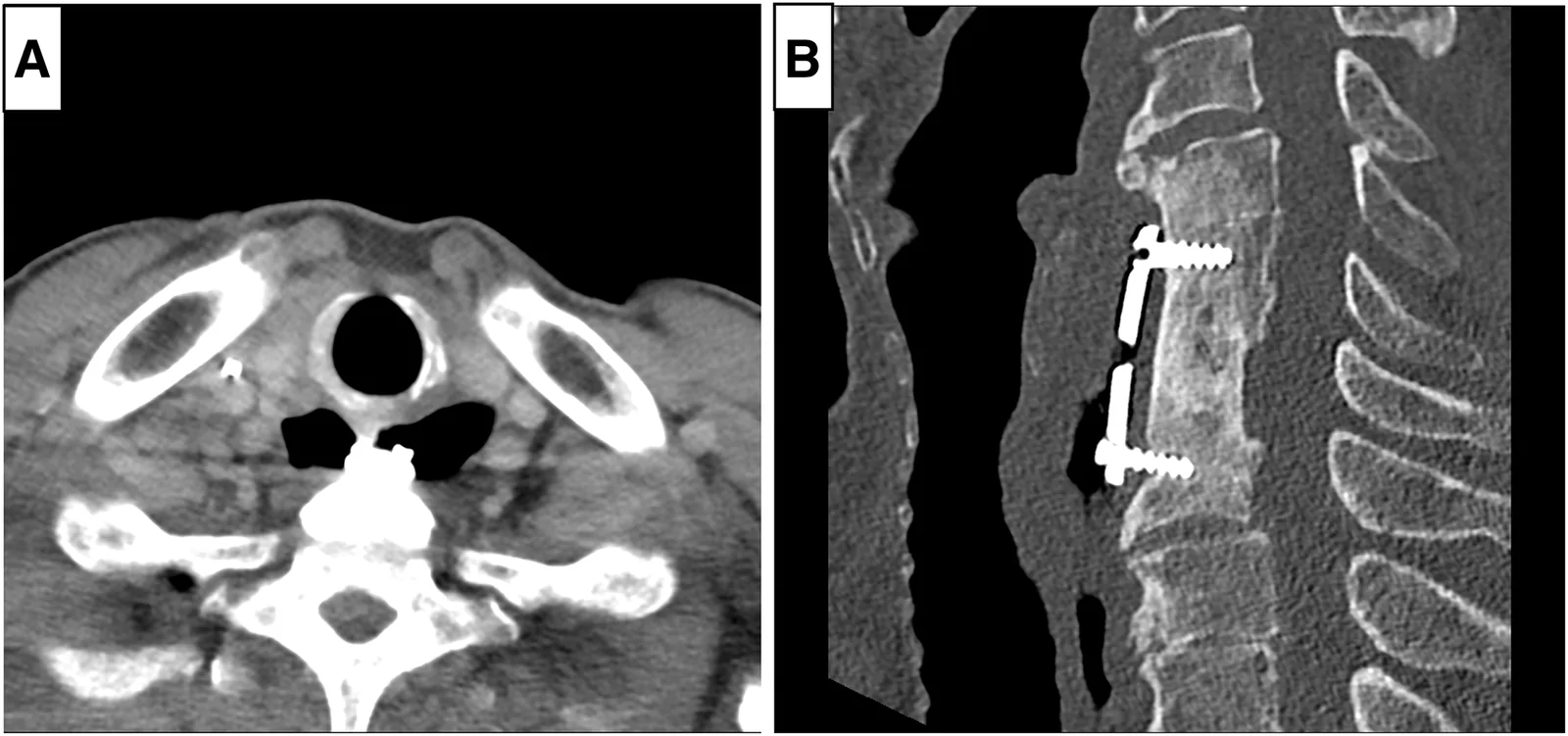
CT examination. (**A**) A metallic plate is present in the dilated cervical esophagus. (**B**) The 5th to 7th cervical spine was fixed by bone screwing with a metallic plate.

**Fig. 3:**
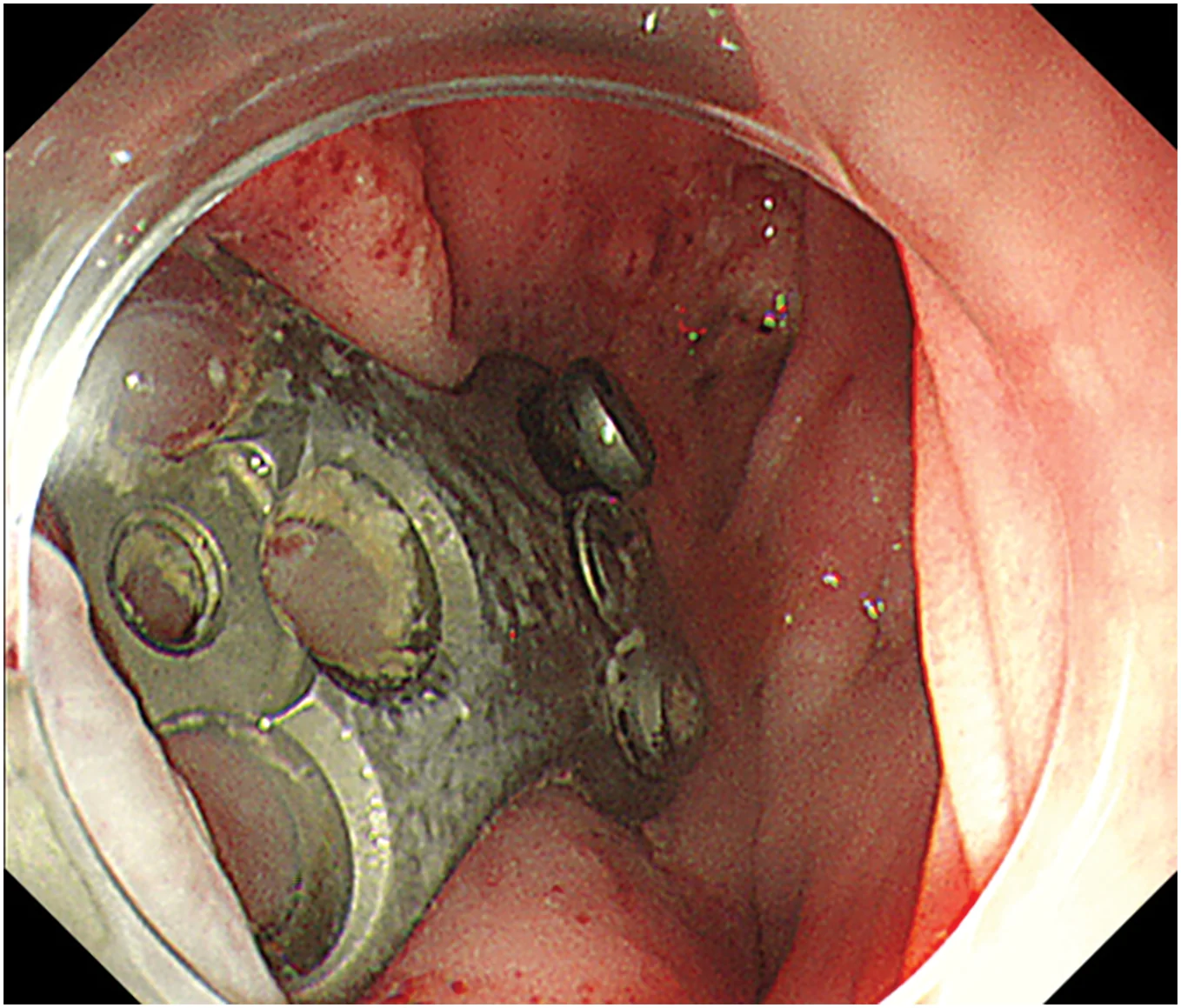
Endoscopic examination shows a metallic plate in the posterior wall of the cervical esophagus.

**Fig. 4:**
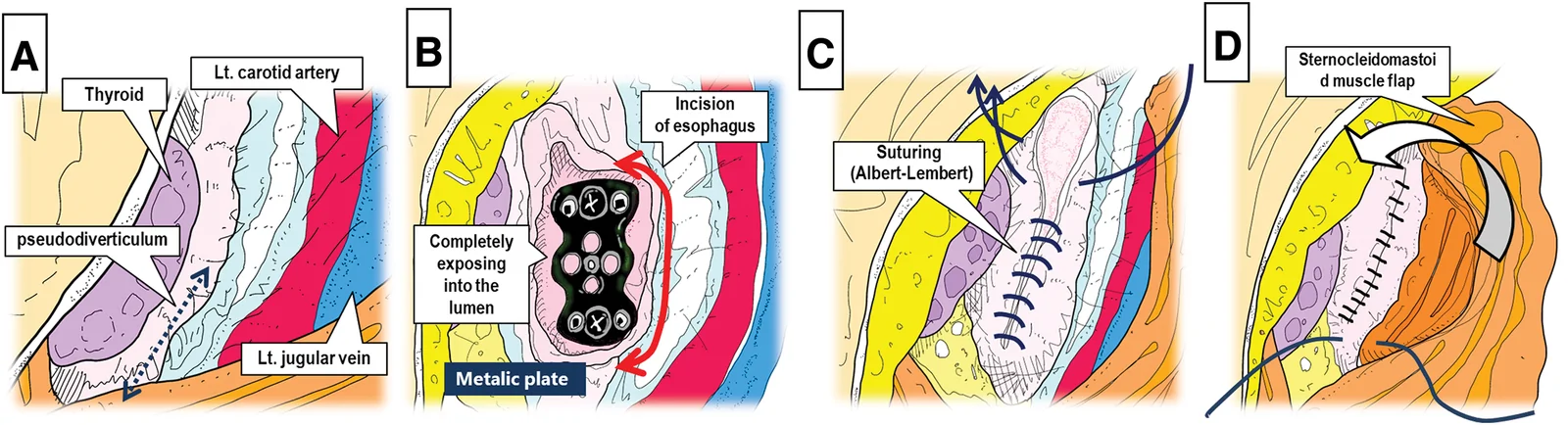
Schema of the performed surgical procedure. (**A**) Cervical esophagus with a cervical esophageal diverticulum adhered to the cervical spine. (**B**) A metallic plate was found to be completely exposed to the pseudodiverticulum in the cervical esophagus. (**C**) After opening the posterior wall of the esophagus, the metallic plate was successfully removed, and the pseudodiverticulum was resected and sutured. (**D**) Subsequently, the suturing site was reinforced using an SCM flap. SCM, sternocleidomastoid muscle

**Fig. 5:**
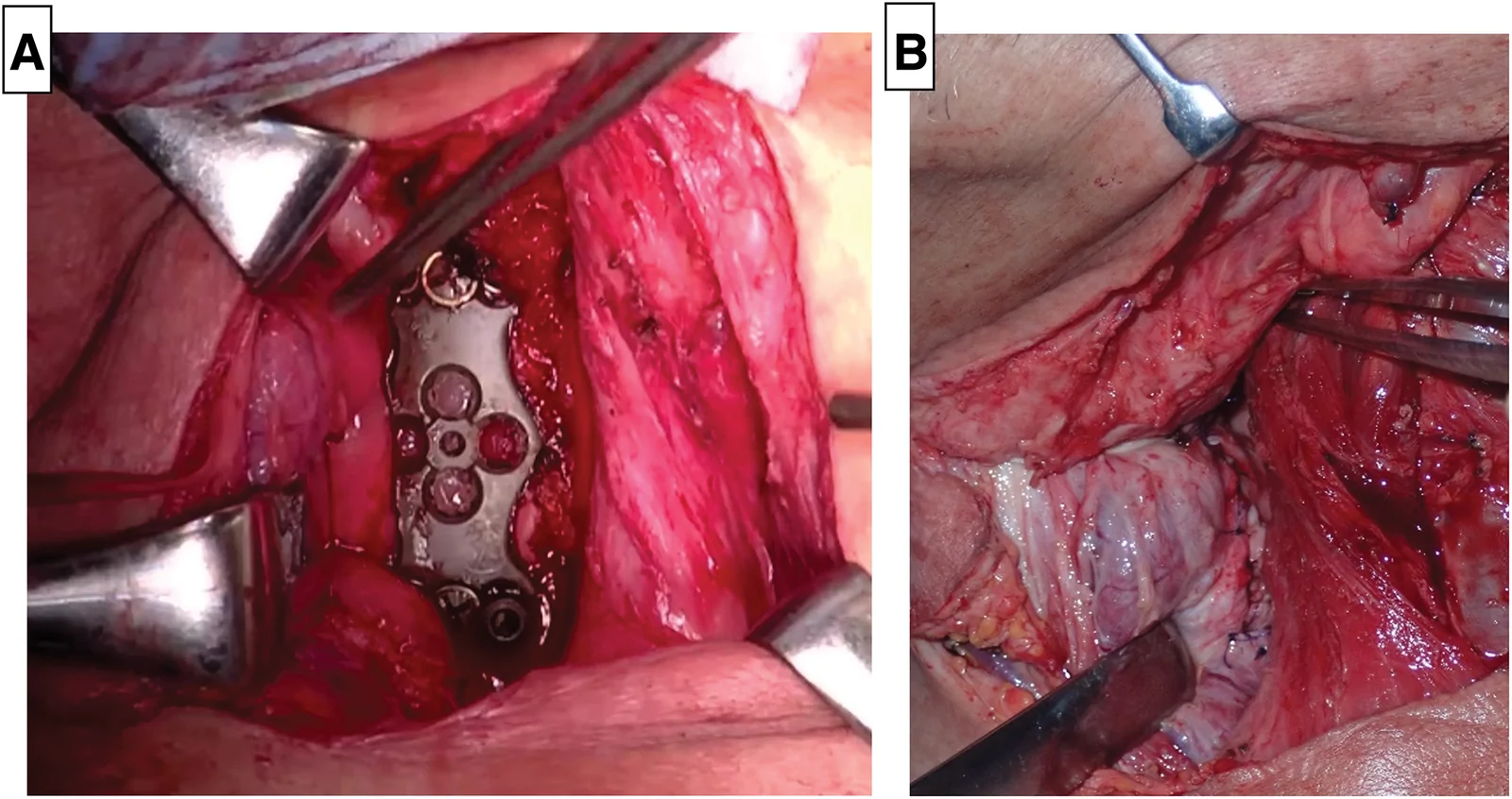
Intraoperative findings. (**A**) The metallic plate was fully exposed to the lumen of the cervical esophagus. (**B**) The sutured cervical esophagus was reinforced with an SCM flap. SCM, sternocleidomastoid muscle

A 63-year-old man who had undergone ACDF 20 years previously was admitted to a local hospital with fever, dysphagia, and neck pain. Cervical spine radiographs revealed an anterior cervical plate spanning the C5–C7 levels (**Fig. 1**). CT revealed a metallic plate along the anterior C5–C7 vertebral bodies with intraluminal migration into the dilated esophagus (**Fig. 2A**). The C5–C7 segment exhibited solid bony fusion (**Fig. 2B**). No evidence of abscess formation was observed in the neck or the mediastinum. Endoscopic study showed a separate orifice near the cervical esophageal inlet. This orifice led to a blind-ended cavity that was distinct from the true esophageal lumen, and the metallic plate was exposed within this cavity (**Fig. 3**). The patient was diagnosed with esophagitis caused by hardware migration into the cervical esophageal diverticulum. The patient was then transferred to Nagasaki University Hospital for surgical treatment. During surgery, an oblique incision was made along the anterior border of the left and right SCMs. The cervical esophagus was dissected from the medial aspect of the left and right common carotid arteries (**Fig. 4A**). The anterior cervical region was reached without damaging the dilated esophagus. The metallic plate was fully exposed to the lumen of the cervical esophageal diverticulum, which had formed between the plate and the posterior esophageal wall (**Figs. 4B**, **5A**). After opening the posterior wall of the esophagus, the metallic plate was removed from the spine. The migrated implant was an ATLANTIS anterior cervical plate made of titanium alloy. The screws had loosened and partially backed out; therefore, they were removed using the dedicated screwdriver, and the plate was removed without difficulty. The esophageal diverticulum was then resected. The posterior esophageal wall was closed with 2-layer interrupted Albert–Lembert sutures using 4-0 polydioxanone (PDS; Ethicon, Bridgewater, NJ, USA) (**Figs. 4C**, **5B**). Preoperative spinal imaging confirmed solid fusion and segmental stability. Therefore, no supplementary fixation was performed after plate removal, and the posterior esophageal wall was reinforced using a left SCM flap (**Fig. 4D**). The SCM flap showed good color and no macroscopic signs of ischemia during the operation. Histological examination of the resected wall of the diverticulum revealed the mucosa and submucosa, but not the muscular layer. Therefore, a pathological diagnosis of pseudodiverticulum formation within the cervical esophagus was established. The patient recovered uneventfully and was discharged after radiologic confirmation of unhampered passage of contrast medium and no signs of leakage. Two years after surgery, the patient was able to eat a regular diet without any anatomical limitations. The CT revealed no dilation of the cervical esophagus.

## DISCUSSION

Hardware migration into the esophagus after ACDF is rare but serious and can cause perforation, infection, obstruction, and deep-neck or mediastinal abscess.^7)^ The esophagus, which is susceptible to intrusion by foreign entities, may consequently develop complications, including perforation, infection, and obstruction. Symptoms indicative of hardware migration, such as dysphagia, pain, or emesis post-ACDF, require prompt consideration of diagnostic modalities such as radiography or endoscopy to confirm the presence and facilitate strategic removal of the migrated object, whether through endoscopic retrieval or surgical extraction.^8)^ Notably, this is a rare but potential delayed complication of ACDF surgery.

We summarized reported delayed esophageal or pharyngoesophageal complications associated with cervical hardware after anterior cervical spine surgery (**Table 1**).^1,3,4,7,9–32)^ The time from the initial surgery to diagnosis varied widely, from several months to more than 10 years. Only 3 cases, including the present case, have been reported in which symptoms developed more than 20 years after ACDF. Although clinical symptoms were not listed separately in the table because they were largely similar among reports, most patients presented with dysphagia, neck pain, fever, or signs of cervical infection. Most patients required removal of the exposed or migrated hardware and repair of the esophageal or pharyngoesophageal defect. Many repairs were reinforced with a vascularized tissue flap, such as an SCM muscle flap or another regional flap.

**Table 1:** Reported delayed esophageal complications associated with cervical hardware after anterior cervical spine surgery

Author	Year	Cases	Age	Sex	Time from surgery to diagnosis	Diverticulum	Treatment	Muscle flap	Outcome	Reference
Witwer and Resnick	2003	1	67	Male	4 years	No	Surgery	SCM muscle flap	Not reported	^9)^
Zdichavsky et al.	2004	1	50	Male	10 months	No	Surgery	SCM muscle flap	Improved	^10)^
Vrouenraets et al.	2004	1	74	Female	10 months	No	Surgery	None or not reported	Improved	^11)^
Summers et al.	2007	1	43	Female	2 years	Yes	Surgery	None or not reported	Fatal	^12)^
Joanes and Belinchón	2008	1	31	Male	3 years	Yes	Surgery	None or not reported	Improved	^13)^
Solerio et al.	2008	1	41	Male	7 years	Yes	Surgery	SCM muscle flap	Improved	^4)^
Dakwar et al.	2009	5	Mean 45	Female, 2; male, 3	1, 7, 24, and 27 months; 6 years	No	Surgery	4 SCM muscle flaps and 1 radial forearm free flap	All recovered; 1 required multiple surgeries	^14)^
Tian et al.	2011	1	31	Male	7 years	Yes	Surgery	Sternohyoid and omohyoid muscle flaps	Improved	^1)^
Almre et al.	2014	1	53	Male	18 years	Yes	Surgery	None	Improved	^15)^
Sadrizadeh et al.	2015	2	24; 46	Female; male	Case 1: 7 years; Case 2: not confirmed	Yes	Surgery	None or not reported	Improved	^7)^
Yang et al.	2015	1	63	Male	8 years	No	Surgery	SCM muscle flap	Improved	^16)^
Lee et al.	2015	2	55; 65	Female; male	2 months; 11 months	No	Surgery	local muscle flap in 1 case	Improved	^17)^
Kang et al.	2017	5	Mean 59	Female, 1; male, 4	14, 8, and 10 years; 3 and 9 months	No	Surgery/Observation	None or not reported	4 recovered; 1 died	^18)^
Volkow-Fernández et al.	2019	1	48	Female	4 years	Yes	Surgery	None or not reported	Improved	^3)^
Gibson et al.	2020	1	80	Male	15 years	Yes	Surgery	Supraclavicular artery island fascial flap	Not reported	^19)^
Shah et al.	2021	1	47	Female	9 months	No	Surgery	Anterolateral thigh free flap	Improved	^20)^
Morita et al.	2021	1	51	Male	20 years	Yes	Surgery	None	Improved	^21)^
Zakko et al.	2022	1	51	Male	12 years	Yes	Surgery	Supraclavicular artery island flap	Not reported	^22)^
Sharma and Sieg	2022	1	60	Male	20 months	No	Stent	None	Improved	^23)^
Yahanda et al.	2022	9	Mean 58	Female, 3; male, 6	Mean 7.5 years	No	Surgery	Rotational flap	Not reported	^24)^
Frankel et al.	2023	1	76	Male	7 months	No	Surgery	SCM muscle flap	Successful treatment	^25)^
Inomata et al.	2023	1	57	Female	Over 3 years after last anterior cervical surgery	No	Surgery	None	Not reported	^26)^
Prasad et al.	2024	7	Median 49	Female, 2; male, 5	Median 38 months; range 5–93 months	No	Surgery	None or not reported	6 recovered; 1 died	^27)^
Luo et al.	2024	5	Mean 44	Female, 2; male, 3	Average 4.9 years	No	Surgery	SCM muscle flap	All recovered	^28)^
Park et al.	2018	1	72	Male	20 years for first perforation; 25 years for recurrence	No	Surgery	None or not reported	Improved	^29)^
Luo et al.	2025	1	50s	Female	10 years	Yes	Surgery	SCM muscle flap	Improved	^30)^
Lackland et al.	2025	1	69	Male	5 years	No	Surgery	None	Died 5 days postoperatively	^31)^
Tharakan et al.	2026	1	24	Male	68 months	No	Surgery	Pectoralis major muscle flap	Improved	^32)^
Present case	2026	1	63	Male	20 years	Yes	Surgery	SCM muscle flap	Improved	—

SCM, sternocleidomastoid muscle

Reported factors associated with delayed esophageal injury or implant migration after ACDF include screw loosening or backout, hardware extrusion, implant displacement, chronic compression or friction against the posterior esophageal wall, infection, and pseudarthrosis.^6,18,21,24)^ Our patient had plate elevation and caudal screw backout. Chronic mechanical irritation between the plate, screws, and posterior wall of the cervical esophagus was therefore considered the main factor leading to pseudodiverticulum formation and plate migration.

The subclinical migration probably progressed gradually over several years. Chronic pressure and friction between the cervical plate and the posterior esophageal wall may have caused gradual pseudodiverticulum formation. The presenting symptoms were likely triggered by local inflammation or infection of the pseudodiverticulum after exposure to the plate.

Endoscopy showed a separate blind-ended cavity near the cervical esophageal inlet, and the plate was exposed inside this cavity. In addition, histological examination of the resected wall showed mucosa and submucosa but no muscular layer. These findings supported the diagnosis of a pseudodiverticulum rather than a true diverticulum or simple full-thickness esophageal necrosis.

**Fig. 6:**
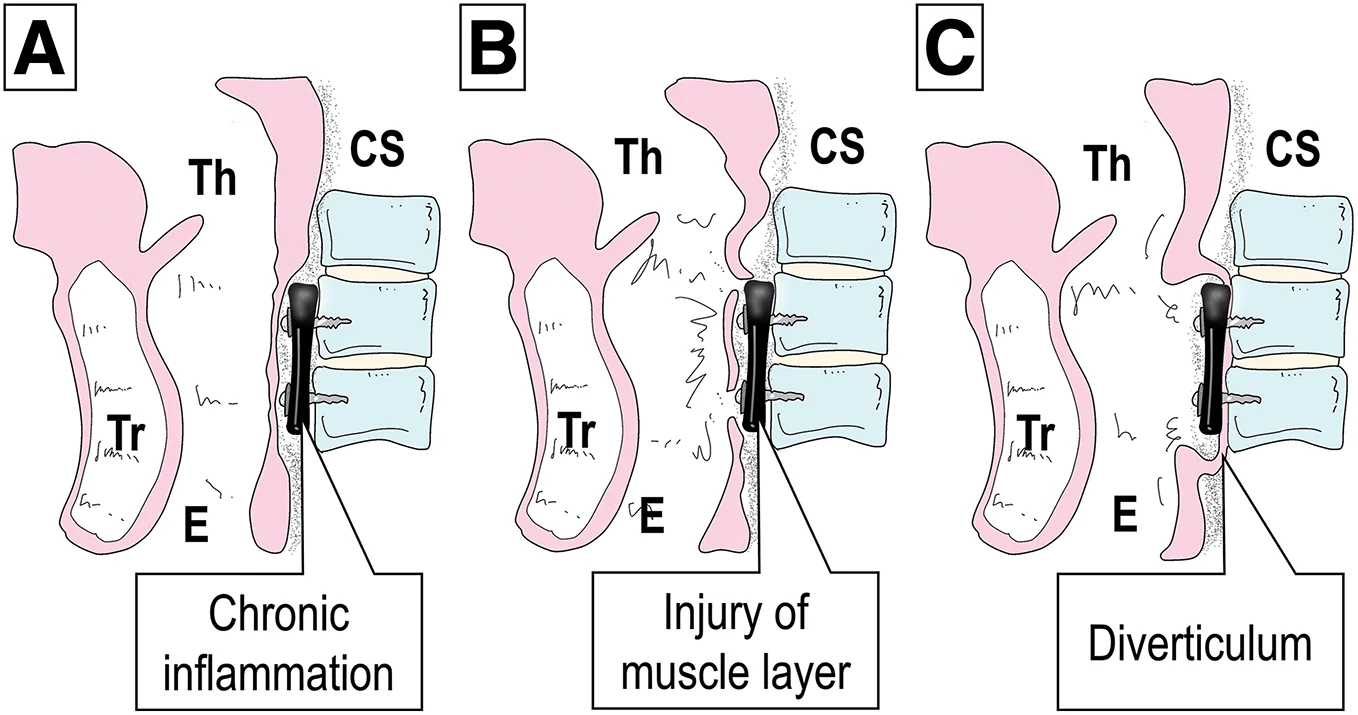
The hypothesis of metallic plate migration in the cervical esophagus. (**A**) Chronic friction between the posterior wall of the esophagus and the metallic plate occurs for long periods after ACDF. (**B**) Traction leads to the development of a pseudodiverticulum in the cervical esophagus. (**C**) Migration of the metallic plate in the esophageal pseudodiverticulum gradually progresses. ACDF, anterior cervical discectomy and fusion; CS, cervical spine; E, esophagus; Th, throat; Tr, trachea

Pseudodiverticulum formation after anterior cervical spine surgery has been attributed to chronic mechanical irritation, pressure, and traction between cervical hardware and the pharyngoesophageal wall.^7,12,13)^ Unlike a true diverticulum, a pseudodiverticulum does not contain all layers of the esophageal wall. In the present case, the blind-ended cavity was distinct from the true esophageal lumen and lacked a muscular layer on histological examination.^33)^ Traction-type diverticulum formation substantiated by dense fibrosis linking the cervical hardware to the posterior pharyngoesophageal wall has been previously reported; however, complete metallic plate migration remains exceedingly uncommon.^7,12,13)^ In this case, such migration likely incited pseudodiverticulum formation through traction forces attributable to fibrotic scarring between the esophagus, prevertebral tissue, and the cervical plate (**Fig. 6**).

The main treatment goals in symptomatic hardware migration are removal of the exposed or migrated implant, closure of the esophageal defect, and prevention of leakage or recurrent injury. Solid bony fusion and segmental stability were confirmed before surgery; therefore, no additional spinal fixation was required after plate removal. After resection of the pseudodiverticulum, the posterior esophageal wall was closed with 2-layer interrupted sutures and reinforced with an SCM muscle flap. Flap reinforcement is important because cervical esophageal leakage can lead to severe complications, including deep-neck abscess, mediastinitis, osteomyelitis, aspiration pneumonia, and sepsis. This approach is consistent with Harman et al., who reported that the SCM flap is commonly used for reinforcement of cervical esophageal injuries.^33)^ The use of flaps in wound reconstruction is supported by several key factors, including provision of a physical barrier to protect the underlying structures and delivering a rich arterial blood supply to the wound bed, which is critical for promoting wound healing and enhancing the local administration of antibiotics.^34)^

The SCM flap is ideal for small perforations because it can be harvested in the same surgical field as head and neck surgery and is minimally invasive.^35,36)^ In our patient, the SCM flap showed good color and no macroscopic signs of ischemia during the operation, suggesting sufficient blood flow for reinforcement. Lee et al. proposed that when the SCM flap is insufficient, the perforation is large (>2 cm), or the vertebral defect is large (>5 mm), the pectoralis major muscle flap should be used.^37)^ When the SCM flap is not feasible or the defect is larger, the latissimus dorsi flap is used as an alternative and is designed to provide sufficient reach. In general, pedicled regional flaps are the most reliable reconstruction methods. Free flaps can cover large esophageal defects; however, the risk of thrombosis is higher in patients with chronically inflamed neck veins. Although contralateral venous anastomosis is possible, it may endanger the recurrent laryngeal nerve.^38,39)^

This case shows that delayed esophageal complications can occur even 20 years after ACDF. In patients with a history of ACDF who present with dysphagia, fever, neck pain, or a cervical infection, hardware migration and esophageal injury should be considered. Complete implant removal, secure esophageal closure, and reinforcement with a vascularized flap may provide good outcomes when spinal stability is confirmed.

## CONCLUSIONS

We report a rare case of complete migration of an anterior cervical plate into a cervical esophageal pseudodiverticulum 20 years after ACDF. Implant removal, direct esophageal closure, and reinforcement with an SCM muscle flap achieved a favorable outcome. This case highlights the need to consider delayed hardware migration in patients with a history of ACDF who present with dysphagia, fever, or neck pain.

## DECLARATIONS

### Availability of data and materials

The data that support the findings of this study are available from the corresponding author upon reasonable request.
